# Microbiota-dependent regulation of costimulatory and coinhibitory pathways via innate immune sensors and implications for immunotherapy

**DOI:** 10.1038/s12276-023-01075-0

**Published:** 2023-09-11

**Authors:** Joon Seok Park, Francesca S. Gazzaniga, Dennis L. Kasper, Arlene H. Sharpe

**Affiliations:** 1 Department of Immunology, Blavatnik Institute, Harvard Medical School, Boston, MA 02115 USA; 2 Department of Pathology and Center for Cancer Research, Massachusetts General Hospital, Charlestown, MA 02129 USA; 3 Department of Pathology, Harvard Medical School, Boston, MA 02115 USA

**Keywords:** Mucosal immunology, Innate immune cells

## Abstract

Our bodies are inhabited by trillions of microorganisms. The host immune system constantly interacts with the microbiota in barrier organs, including the intestines. Over decades, numerous studies have shown that our mucosal immune system is dynamically shaped by a variety of microbiota-derived signals. Elucidating the mediators of these interactions is an important step for understanding how the microbiota is linked to mucosal immune homeostasis and gut-associated diseases. Interestingly, the efficacy of cancer immunotherapies that manipulate costimulatory and coinhibitory pathways has been correlated with the gut microbiota. Moreover, adverse effects of these therapies in the gut are linked to dysregulation of the intestinal immune system. These findings suggest that costimulatory pathways in the immune system might serve as a bridge between the host immune system and the gut microbiota. Here, we review mechanisms by which commensal microorganisms signal immune cells and their potential impact on costimulation. We highlight how costimulatory pathways modulate the mucosal immune system through not only classical antigen-presenting cells but also innate lymphocytes, which are highly enriched in barrier organs. Finally, we discuss the adverse effects of immune checkpoint inhibitors in the gut and the possible relationship with the gut microbiota.

## Costimulatory and coinhibitory pathways: bridge between gut microbiota and immunotherapy

"Microbiota-dependent regulation of costimulatory and coinhibitory pathways via innate immune sensors and implications for immunotherapy," highlights the role of commensal microbes in immune cell regulation. Researchers have demonstrated that certain human bacteria and their products can influence abundance and differentiation of immune cells in the colon, small intestine, and other organs. The host immune system can sense the presence of certain gut microbes through evolutionarily conserved proteins, which can bind to specific bacterial molecules. The host-microbiota interaction leads to changes in expression of costimulatory and coinhibitory molecules, shaping immunity in various contexts. The gut microbiota is linked to responses to cancer immunotherapy and its adverse effects via costimulatory and coinhibitory pathways. Understanding these immunomodulatory mechanisms could lead to the development of therapies for a wide range of diseases.

## Introduction

Our body is a living place for not only host cells but also numerous microorganisms, collectively called the microbiota. Most commensal microbial colonization is symbiotic, benefiting the host. These microbes colonize barrier organs, including the intestines, where the host immune system plays an important role in balancing mucosal immunity. Recent studies have shown that the gut microbiota is closely associated with immune-related diseases such as inflammatory bowel disease (IBD), persistent antibiotic-induced colitis, atopic asthma, type 1 diabetes and autoimmunity^1^. Commensal microbes can regulate various immune cell types. Intestinal T-cell differentiation is significantly impacted by specific microbes. Commensal microbes can be processed by dendritic cells to induce IgA production^2^. The gut microbiota also regulates the cellular diversity and landscape of innate lymphoid cells (ILCs)^3^.

How the gut microbiota shapes immune cells has been an active field of research. Multiple pattern recognition receptors (PRRs), including Toll-like receptors (TLRs) and NOD-like receptors (NLRs), can transduce microbe-derived signals to immune cells. These innate sensing mechanisms can drive large changes in the mucosal immune system. Thus, signaling mediators and pathways connecting bacterial molecules to immune cell differentiation can play a pivotal role in host-microbiota interactions.

Unlike pathogenic infections, which induce inflammation and disease^4^, commensal gut microbes shape immunity in more subtle ways. Certain commensal bacteria may impact the frequency of immune cell types^5^ or impact immune cell function^6,7^, which, while not directly causing inflammation, may predispose the host to or protect the host from a variety of diseases. This review will focus specifically on commensal-immune signaling.

## Gut microbiotas shape immunity by impacting cell frequencies

Perhaps the most striking effect of the gut microbiome on the immune system can be seen with the naked eye. Germ-free (GF) mice, which are raised in sterile isolators and devoid of all microbes, have visibly smaller Peyer’s patches^8^ and fewer T cells in their Peyer’s patches and mesenteric lymph nodes (MLNs)^9^. The impact of specific human bacteria on immune cell frequencies was investigated by mono-colonizing GF mice with 53 different human commensal bacteria and analyzing immune cell frequencies in the colon lamina propria, small intestine lamina propria, Peyer’s patches, mesenteric lymph nodes, and spleen^5^. At baseline, the frequencies and subset compositions of some immune cell types, such as RORγt+ Tregs or macrophages in the colon, are changed and highly dependent on the presence of microbes, whereas other immune cell types do not vary with colonization^6,7^. In other cases, the impact of colonization with specific bacteria is only observed in the context of disease settings^10,11^.

## Scratching the surface of signaling by commensal organisms

It has been known for almost twenty years that commensals, and not just pathogens, signal through host TLRs^12^. Our understanding of how the host immune system distinguishes signals from commensals and pathogens and interprets signals from commensal organisms to maintain homeostasis and modulate disease is in its infancy. Here, we explore the known commensal microbe-immune signaling pathways and their impacts on health and disease. First, we will explore how secreted bacterially derived metabolites impact immune function. Next, we will discuss bacterial signaling, including that by bacterial surface molecules and other bacterial molecules that could be on surface, membrane, or in unknown locations.

## Microbial metabolites

The gut microbiota produces numerous metabolites that can affect host immune function^13^. Although the exact immunomodulatory molecules have not been identified, gut microbiota metabolites can activate innate lymphoid cells (ILCs); the interactions between the gut microbiota and ILCs have been reviewed elsewhere^14–16^. In the mucosa, where commensal microbes reside, one of the key immune subsets is ILCs, which are distinct from conventional lymphocytes^16^. Similar to other lymphocytes, ILCs develop from common lymphoid progenitors (CLPs). ILCs can differentiate into five major subsets including NK cells, ILC1s, ILC2s, ILC3s, and LTi cells. ILCs play a role in modulating adaptive immunity by interacting with T cells in the intestines and other barrier organs. The lineage specification of ILCs resembles that of helper T-cell differentiation programs, and each group of ILCs shares functional similarities and transcriptional programs with T helper cell counterparts. For example, ILC1s express T-bet and IFN-γ like TH1 cells; ILC2s express GATA-3, IL-5 and IL-13 like TH2 cells; and ILC3s express RORγt and IL-17 like TH17 cells^16^.

Gut commensals can activate ILC3s. CX3CR1 mononuclear phagocytes sense microbial signals in a MYD88-dependent manner to induce IL-23 and IL-1β, which leads to increased release of IL-22 and GM-CSF by ILC3s^17–19^. *Lactobacillus* species can metabolize dietary tryptophan into AHR ligands that stimulate ILC3s^20^. This microbiota-ILC3/TH17 crosstalk plays an important role in intestinal barrier homeostasis, colitis, and intestinal infection^17,21^.

The dependence of ILC2 activity on the gut microbiota is less clear, but ILC2s can also be activated by components of the microbiota. The microbial metabolite succinate signals through Tuft cells in the gut to induce IL-25, which activates ILC2 production of IL-13 and IL-5, leading to increased goblet cell and mucous production^14,15,22,23^. ILC2s play an important role in immune homeostasis in the lung and the response to infection, yet how ILC2s sense microbial signals and what microbial signals impact ILC2 function remain unclear^16^.

Short-chain fatty acids (SCFAs), produced by many members of the gut microbiota, can signal through G-protein coupled receptors (GPRs) on epithelial cells to induce regulatory T cells. Gut microbes can metabolize bile acids in the colon to induce RORγt+ Tregs by signaling through vitamin D receptor (VDR)^24^, which plays an important role in protecting against colitis. Other gut microbe-derived bile acid metabolites inhibit TH17 signaling and enhance Treg signaling^25^. Microbial bile acids may influence bariatric surgery outcomes and obesity-associated comorbidities^26^.

Taken together, these examples illustrate how microbiota-derived molecules can influence immune function. Further understanding of the immunomodulatory mechanisms of the microbiota could be beneficial for developing therapies for a wide range of diseases.

## Bacterial signaling molecules

### Bacteroides species

**Fig. 1:**
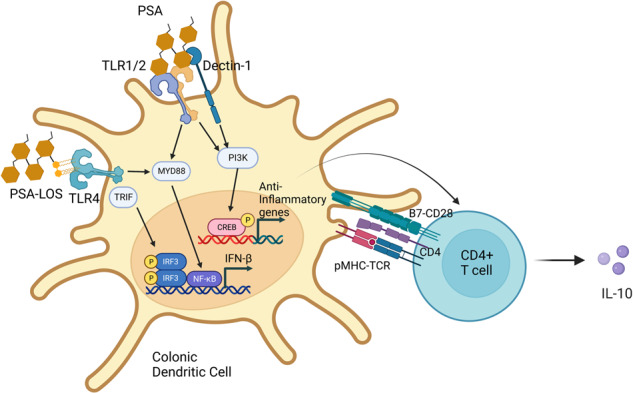
Bacterial PSAs transduce signals through innate receptors to modulate immune responses. TLR1/2 and Dectin-1 mediate PSA-induced PI3K activation, which leads to CREB-dependent transcription of anti-inflammatory genes in dendritic cells. This signaling pathway might drive dendritic cells to differentiate T cells into IL-10-producing cells. The LOS portion of PSA can bind to TLR4 and activate the MyD88-dependent NF-kB signaling pathway and the TRIF-dependent IRF3 signaling pathway, leading to the transcription of proinflammatory cytokines (e.g., IFN-β).

The phylum Bacteroidetes is one of the two most prominent phyla that make up the human gut microbiome and is one of the early colonizers of the infant gut^27,28^. Therefore, it is unsurprising that Bacteroides species have multiple immunomodulatory effects. For example, polysaccharide A (PSA) isolated from the surface of the gut commensal organism *Bacteroides fragilis* contains a lipid A portion and a polysaccharide portion that signal through TLR1/2 heterodimers in conjunction with Dectin-1 to initiate phosphoinositide 3-kinase (PI3K) pathway signaling, leading to CREB-dependent expression of anti-inflammatory genes such as IL-10 in dendritic cell-CD4^+^ T-cell co-cultures^9^ (Fig. 1). PSA treatment protects against disease in mouse models of colitis and experimental autoimmune encephalitis (EAE)^29–31^. Lipooligosaccharides (LOS) on the outer membranes of *B. fragilis* and other Bacteroides species can signal through TLR4 on dendritic cells to induce interferon-β secretion, and treatment with PSA (containing the LOS) protects against murine vesicular stomatitis virus (VSV) infection^32^.

PSA and LOS are not the only molecules from *B. fragilis* that induce immune signaling. *B. fragilis* also expresses alpha galactosylceramides (αGCs)^33–35^. αGCs complex with CD1d and the invariant natural killer T-cell (iNKT) cell receptor and stimulate iNKT cells to produce IL-2 and IFNγ^33,35^. The structure of αGCs impacts their immunomodulatory effects. For example, the synthetic iNKT cell agonist KRN7000, which robustly stimulates IL-2 production in APC-NKT cell co-cultures^33^, has a longer N-acyl chain than αGCs and a hydroxyl group at C4 of the sphinganine base^35^. These differences could at least partially explain the differences between how KRN7000 and αGCs modulate immune function. Stimulation of NKT cells with KRN7000 results in both IL-4 and IFN-γ release, but stimulation with synthetic versions of KRN7000 that have shorter N-acyl chains bias toward IL-4 release. Removal of the hydroxyl group at C4, on the other hand, results in loss of affinity for the TCR^34^. Additionally, the sphingosine chain branching of αGCs affects their immunomodulatory effects on gene expression and cytokine release, with branched chain αGCs stimulating more IL-2 production than straight chain αGCs^36^. Whether the lack of virulence factors in commensal strains, the ability of PSA and αGCs to have both immune stimulatory and inhibitory effects^32,37^, or some yet to be determined factors distinguish commensal strains of *B. fragilis* from pathogens that signal through TLRs is unclear.

### *Akkermansia mucinophila*

Another gram-negative bacterium, *Akkermansia mucinophila*, is a human gut commensal that is considered beneficial because it is negatively correlated with inflammatory bowel disease and type 2 diabetes and is associated with an antitumor response to anti-PD-1 therapy in melanoma patients^38,39^. Recently, a phospholipid isolated from the cell membrane of *A. mucinophila* was shown to signal through TLR1/2 heterodimers to induce TNFα and IL-6 signaling in BMDCs. Notably, this induction was weaker than that following treatment with LPS (TLR4 agonist) or Pam3CSK4 (TLR2 agonist) and did not induce other cytokines, such as IL-23A or IL-12β, that are induced upon treatment with LPS or Pam3CSK4^40^. This more specific signaling response could at least partially be due to the differences in structure between the *A. mucinophila* phospholipid and traditional TLR agonists but could also be due to other immunomodulatory effects.

### Segmented filamentous bacteria

Segmented filamentous bacteria (SFB), commensal bacteria isolated from Taconic mice, specifically colonize the ileum, induce TH17 cells and stimulate type 3 innate lymphoid cells (ILC3s). SFB colonization stimulates epithelial cells in the gut to produce serum amyloid A (SAA 1 and SAA2), which promotes TH17 cells to secrete IL-17 and ILC3s to secrete IL-22^18^. SFB colonization protects against *Citrobacter rodentium* infection^41^ and can also promote autoimmunity^42^. Although the immunomodulatory molecules produced by SFB are unknown, TH17 induction is dependent on MHCII expression by dendritic cells, suggesting that SFB antigens presented by intestinal dendritic cells can drive TH17 differentiation^41,43^.

## Modulation of costimulation by innate sensors

The two-signal model^44^ for T-cell activation was proposed to explain the requirement for a second signal in addition to the T-cell receptor (TCR) signal for T-cell activation^45^. Intensive studies of costimulation have advanced our understanding of these second signals. We now appreciate that these second signals not only regulate the initial activation of naïve T cells but also control effector, memory, and regulatory T cells. In addition, there are both positive secondary signals (costimulatory) that stimulate T-cell responses and negative secondary signals (coinhibitory) that inhibit T-cell responses^46^. Costimulatory and coinhibitory molecules can be expressed not only on antigen-presenting cells but also on nonhematopoietic cells, which may enable these molecules to regulate T-cell responses locally in specific tissues.

Early studies identified interactions between CD28 and its ligands B7-1 (CD80) and B7-2 (CD86) as the major secondary signal (costimulatory signal) required for initial T-cell activation. Upon TCR engagement of cognate peptide-MHC, ligation of CD28 on T cells by B7-1 or B7-2 on antigen-presenting cells (APCs) provides ‘signal 2’ that fully activates T cells. Costimulatory and coinhibitory pathways have been implicated in many disease contexts, primarily related to T-cell-mediated immune responses. These pathways not only regulate the adaptive immune system but also, as more recently reported, the functions of these molecules have expanded to the regulation of innate cells, including macrophages, dendritic cells, and innate lymphocytes. Therefore, stimuli that control the surface expression of these costimulatory and coinhibitory receptors and their ligands can shape various types of immune responses. The expression of costimulatory and coinhibitory molecules can be modulated by the tissue environment, activation states, cytokines and innate receptor signaling^47^.

The most well-known family of costimulatory and coinhibitory molecules is the B7 superfamily, which includes B7-1, B7-2, ICOSL (CD275, B7RP-1, B7-H2), PD-L1 (CD274, B7-H1), PD-L2 (CD273, B7-DC), B7-H3 (CD276), and B7-x (B7-H4, B7S1)^48^. B7 family members belong to the immunoglobulin superfamily and contain extracellular IgV and IgC domains. These molecules can be transmembrane or have a glycosylphosphatidylinositol (GPI)^49^ anchor. B7-1 and B7-2 bind to CD28 and cytotoxic T-lymphocyte-associated protein 4 (CTLA-4, CD152), but the binding affinity of B7-1 and CTLA-4 is much higher than that of B7-1 and CD28^50,51^. ICOSL binds to ICOS (CD178)^52^. PD-L1 and PD-L2 share PD-1 as a binding partner^53^, but each has a second unique binding partner. PD-L1 also binds B7-1^54^, and PD-L2 binds repulsive guidance molecule b (RGMb)^55^. While the expression of B7-1, B7-2 and PD-L2 is primarily on antigen-presenting cells (APCs), PD-L1, B7-H3 and B7x can also be more broadly expressed on nonhematopoietic cells and tumors^56,57^. The functions of B7 family members and their receptors have been actively investigated and revealed that these pathways can transduce both positive signals and negative signals depending on the context and receptors^58^.

**Fig. 2:**
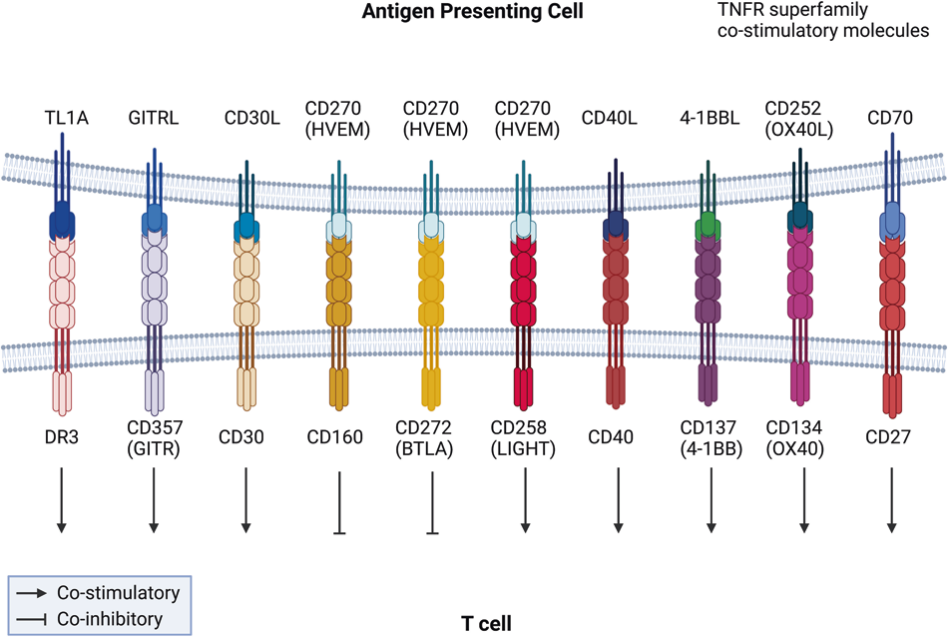
TNFR superfamily costimulatory molecules. TL1A/DR3, GITRL/GITR, CD30L/CD30, HVEM/LIGHT, CD40L/CD40, 4-1BBL/4-1BB, OX40L/OX40 and CD70/CD27 interactions provide costimulatory signals to T cells, while ligation of HVEM with BTLA or CD160 provides coinhibitory signals to T cells.

Another class of costimulatory pathways belongs to the tumor necrosis factor receptor superfamily (TNFR)^59^. These include the OX40 (CD134)/OX40L (CD252), CD40/CD40L, 4-1BB (CD137)/4-1BBL, CD27/CD70, Herpesvirus entry mediator (HVEM, CD270)/LIGHT (CD258)/B- and T-lymphocyte attenuator (BTLA, CD272)/CD160, CD30/CD30L, Glucocorticoid-induced TNFR-related protein (GITR, CD357)/GITRL, and Death domain receptor 3 (DR3)/Tumor necrosis factor-like cytokine 1A (TL1A) pathways^60,61^ (Fig. 2, Table 1). HVEM is widely expressed in all lymphocytes, including resting T and B cells, NK cells, Tregs, monocytes, and dendritic cells (DCs), as well as mesenchymal cells and epithelial cells^62^. LIGHT is expressed in DCs, macrophages, neutrophils, NK cells, ILCs, NKT cells and activated CD4+ and CD8+ T cells but not naïve T cells, Tregs and B cells^63^. BTLA, CD27 and DR3 expression on naïve T cells is low but upregulated upon activation^64,65^. Other members have inducible expression on APCs (e.g., OX40L, 4-1BBL) or T cells (e.g., OX40, 4-1BB)^59^. Most of these pathways are costimulatory for T-cell responses. However, HVEM can transduce coinhibitory signaling when binding to its ligands CD160 or BTLA, in contrast to transducing a costimulatory signal when engaging LIGHT^66^.

**Table 1:** TNFR superfamily costimulatory molecules.

Receptors	Ligands	Signal
OX40 (CD134)	OX40L (CD252)	Costimulatory
CD40	CD40L	Costimulatory
4-1BB (CD137)	4-1BBL	Costimulatory
CD27	CD70	Costimulatory
LIGHT (CD258)	HVEM (CD270)	Costimulatory
CD160		Coinhibitory
BTLA (CD272)		Coinhibitory
CD30	CD30L	Costimulatory
GITR (CD357)	GITRL	Costimulatory
DR3	TL1A	Costimulatory

In addition, other sets of pathways with costimulatory and coinhibitory functions have been identified. These include the T-cell immunoreceptor with Ig and ITIM domains (TIGIT), T-cell immunoglobulin and mucin-domain containing-3 (Tim-3, CD366) and LAG-3 (CD223) pathways. TIGIT shares the ligands CD155 and CD112 with CD226. CD226 promotes T-cell and NK cell immunity^67,68^. TIGIT negatively regulates NK and T-cell responses and has a higher affinity for CD155 than DNAM-1. T-cell immunoglobulin and mucin-domain containing-3 (Tim-3, CD366) is expressed on a variety of immune subsets, including T cells, myeloid cells, NK cells, mast cells and DCs, and has Galectin-9 (Gal-9), phosphatidylserine, Carcinoembryonic antigen cell adhesion molecule 1 (CEACAM-1) and High motility group Box 1 (HMGB1) as ligands. The binding of Tim-3 to phosphatidylserine is crucial for the phagocytosis of apoptotic cells and antigen presentation by DCs^69^. Recent studies have revealed an important role for Tim-3 in DCs in limiting inflammasome activation and showed that loss of Tim-3 in DCs can increase inflammasome activation and promote antitumor immunity^70^. Finally, the coinhibitory receptor LAG-3 is expressed on T cells and NK cells. The known ligands for LAG-3 are MHC class II molecules, fibrinogen-like protein 1 (FGL-1), liver sinusoidal endothelial cell lectin (LSECtin) and galectin-3 (Gal-3)^71–73^. LAG-3/Gal-3 interactions and LAG-3/LSECtin interactions inhibit IFN-γ production by CD8^+^ T cells^72,74,75^. Recent data show that LAG-3 functions as a T-cell signaling disruptor in an MHC class II-independent fashion. During the formation of the immunological synapse, LAG-3 is constitutively associated with the TCR-CD3 complex, where it interacts with the CD4 or CD8 coreceptors. The cytoplasmic tail of LAG-3 regulates the magnitude of TCR-induced signaling through the dissociation of Lck from the CD4 or CD8 coreceptors, resulting in loss of coreceptor-TCR signaling and thereby limiting T-cell activation^76^.

The expression of costimulatory and coinhibitory molecules can be altered by innate sensors such as TLRs, DAMPs and NODs, either as a direct downstream effect of innate receptor signaling or by an indirect effect of cellular activation. In the intestines, immune cells can be exposed to a wide range of bacterial molecules, which can stimulate these cells through these innate receptors. Of note, ligation of innate receptors on APCs often leads to activation and maturation of the cells. Below, we discuss how TLRs, DAMPs and NOD receptors regulate the expression of costimulatory and coinhibitory molecules.

## Toll-like receptors

Among bacteria-derived signaling pathways, the role of TLRs in regulating the expression of B7 family members is well documented. An early study on innate recognition showed that TLR4 stimulation induces B7-1 upregulation^77^. In addition, LPS-induced DC maturation dramatically increases B7-1 and B7-2 expression^78,79^. Remarkably, MyD88, a downstream signaling adaptor of TLR signaling pathways, did not control B7-1 and B7-2 expression, while TLR4-mediated cytokine production was dependent on MyD88^80,81^. However, MyD88 was required for TLR9-induced expression of B7-1 and B7-2, indicating that specific TLR signaling cascades govern costimulatory molecules. Peptidoglycan and lipoteichoic acid-induced TLR2 signaling increase B7-2 expression on DCs^82^. TLRs also regulate ICOSL expression^83^. LPS/TLR4-mediated ICOSL upregulation on macrophages primarily depends on MyD88 and less on TIR-domain-containing adaptor-inducing interferon-β (TRIF). TLR3 activation by poly I:C can also increase ICOSL expression. The TLR2 agonist Pam3CSK4 increases the expression of B7-1, B7-2 and ICOSL on plasmacytoid DCs (pDCs)^83^.

Interestingly, TLRs not only promote costimulatory molecule expression but also induce the expression of coinhibitory molecules. For example, PD-L1 is upregulated upon LPS treatment of macrophages^83^, whereas PD-L2 expression remains remarkably unchanged. Stimulation of BMDCs with *Schistosoma japonicum* antigens elevates PD-L2, not PD-L1, expression in a TLR2-dependent manner^84^. *Schistosoma mansoni* worms, which have lipids capable of transducing signals through TLR2, selectively induce the expression of PD-L1 on macrophages but not other B7 molecules^85,86^. Beyond innate immune cells, TLR2 signaling by heat-killed *Staphylococcus aureus* or Pam3CKS4 can upregulate PD-L1 expression in head and neck squamous cell carcinoma (HNSCC) cells^87^. TLR4 activation drives PD-L1 expression on CD90^+^ colonic myofibroblasts/fibroblasts^88^. For B7-H3, bacterial agonists of TLR2 and TLR4 can induce its expression on human monocytes^89^. LPS upregulates B7x expression on renal tubular cells, podocytes, and glomerular endothelial cells^90^. These studies collectively suggest that modulation of coinhibitory signaling pathways by TLRs might be specific to certain contexts and ligands.

Similar to B7 superfamily members, tumor necrosis factor receptor family (TNFR) members can be upregulated on antigen-presenting cells upon TLR signaling activation. For example, CD40 expression is induced by TLRs^79–81,83^. CD70, the ligand for CD27, is upregulated on BMDCs upon LPS, Poly I:C and CpG treatment^91^. Zymosan, a ligand for TLR2, and LPS treatment induce CD30L expression on DCs but not on macrophages or B cells^92^. LPS can transiently induce GITRL expression on BMDCs and macrophages^93^. Additionally, LPS can induce OX40L upregulation on splenic DCs, which constitutively express OX40L on their surface, and on B cells^94^. BTLA expression can be induced by LPS stimulation of BMDCs^95^. Thus, TLR signaling can induce the expression of TNFR family members on multiple cell types.

TLR stimulation also modulates the expression of other costimulatory ligands. For example, ligands of TLR1/2, TLR3, TLR4, TLR7/8, and TLR9 can induce CD155 expression on the surface of macrophage cell lines, BMDCs, BMDMs and B cells in a MyD88- and/or TRIF-dependent manner^96^. CD155 upregulation by TLR agonists requires NF-kB signaling, not MAPK signaling. Tim-3 expression on peritoneal macrophages is elevated 8 h after LPS treatment and then diminishes^97^. Interestingly, Tim-3 signaling can inhibit TLR4-mediated NF-kB activation. The Tim-3 ligand Gal-9 can be upregulated by TLR3 and TLR4 stimulation in microglia^98^. These examples illustrate how TLR signaling can regulate the expression of a variety of costimulatory and coinhibitory molecules.

Notably, TLR2 signaling itself can act as a costimulator of T-cell activation^99^. Activated T cells express TLR2 and TLR4. Bacterial lipoprotein (BLP), a ligand of TLR2, can enhance TCR-mediated cytokine production, whereas LPS does not induce this effect. TLR1/2 activation of CD8^+^ T cells enhances the expression of 4-1BB, OX40L and GITR in a MyD88-dependent manner^100^, suggesting that TLR2 in T cells may increase T-cell responses by sensitizing T cells to other costimulatory receptors, as well as by directly enhancing T-cell activation as a costimulatory receptor. Additionally, TLR2 costimulation can increase the functionality of antitumor T cells^101^. Perhaps TLR2 may be an ancient form of costimulation by which bacteria can directly influence T-cell activation, but this does not occur through antigen-presenting cells.

## NOD-like receptors

While TLRs can recognize gut microbes extracellularly, nucleotide-binding oligomerization domain-like receptors (NLRs) sense bacterial components inside cells. NOD1 and NOD2 are functionally well described. NOD1 detects γ-D-glutamyl-*meso*-diaminopimelic acid (iE-DAP), and NOD2 recognizes muropeptides from bacterial peptidoglycan^102–104^. NLR signaling can induce the expression of costimulatory and coinhibitory molecules. For example, stimulation of NOD2 with muramyl dipeptide induces PD-L1 and ICOSL on human monocytes^105,106^. Interestingly, such upregulation is not observed for CD80, CD86 and PD-L2^106^. Intravenous injection of FK-565, a ligand of NOD1, can induce upregulation of CD86 and CD40 on CD8α + DCs in mice in vivo^107^. Additionally, in vitro stimulation of BMDCs with NOD ligands increases the expression of CD40, CD70 and CD86^108^. However, CD8α- DCs show minimal changes in expression of these costimulatory molecules, suggesting that NOD1 and NOD2 might alter costimulation capacity in a cell-type specific manner. Collectively, NOD1 and NOD2 appear to activate DCs and increase the expression levels of costimulatory molecules.

While NOD ligands augment the effect of TLR2 on costimulatory molecule expression^108^, several other NLR family members negatively modulate TLR signaling. NLRC3, NLRP6, NLRP12 and NLRX1 inhibit macrophage activation induced by TLR2 and TLR4 agonists^109–112^ and attenuate NF-kB signaling and cytokine expression. Remarkably, the effects of these inhibitory NLRs on costimulatory and coinhibitory molecules remain largely unknown. NLRP3, which can sense bacterial RNAs, can upregulate PD-L1 expression on cancer cells^113,114^. Given that the intratumoral microbiota has emerged as one of the key elements in regulating the tumor microenvironment, antitumor immune responses and metastasis^115–117^, NLRs in the tumor microenvironment may act as a bridge between intratumoral bacteria and immune suppression by modulating coinhibitory molecules/pathways in tumor cells.

## Costimulatory pathways in innate lymphoid cells (ILCs)

Recent studies have shown that ILCs have the capability to present antigens to T cells with costimulation^118^. For example, a subset of ILC3s that lack natural cytotoxicity receptor (NCR) on their surface express (NCR^-^ILC3s) MHC class II and induce antigen-specific T-cell responses^119^. NCR^-^ILC3s can be activated by various TLR and NOD ligands. Notably, they express CD40, CD80 and CD86 upon IL-1β stimulation^119^. In contrast, these costimulatory molecules are expressed at low levels on NCR^-^ILC3s from the small intestine, where the cells interact with the gut microbiota at the steady state^120,121^. During gastrointestinal infection with the helminth *Nippostrongilus brasiliensis*, expression of PD-L1 but not PD-L2 is induced in ILC2s^122^. CD30L, 4-1BBL, CD70 and OX40L are highly expressed on ILC2s in the lung, and OX40L expression can be further induced by IL-33^123^. Interestingly, microbiota-derived NOD2 signaling by muramyl dipeptide can indirectly regulate ILC2s through IL-33 induction in macrophages in mice susceptible to Crohn’s disease^124^. IL-33-activated ILC2s may potentially induce ileitis by modulating costimulation, suggesting a means by which microbiota may indirectly tune ILC-mediated costimulation. In the intestines, the IBD-associated microbiota triggers CX3CR1+ mononuclear phagocytes to produce TL1A, which upregulates OX40L on all ILC3 subsets after engaging its receptor DR3^125^. In a T-cell colitis model, OX40L expression on ILC3s led to increased TH1 differentiation in a DR3-dependent manner. ILC2s express both ICOS and ICOSL, and their ligation is required for ILC2 functions^126^. The HVEM/LIGHT axis also participates in the costimulatory actions on ILCs. ILC3 expression of HVEM is critical for IFN-γ-mediated protection against enteric infection^127^. Collectively, these studies show how costimulatory ligands on ILCs can regulate T-cell-mediated mucosal immunity.

ILCs also express costimulatory and coinhibitory receptors that play a cell-intrinsic role in controlling ILC functions. For example, PD-1 negatively regulates a KLRG-1 + ILC2 population and IL-33-activated ILC2s^128,129^. TNFR2 expression on ILC2s is important for ILC2 function and survival in the lungs^130^. Engagement of GITR with an anti-GITR agonistic monoclonal antibody stimulates ILC2s to secrete TH2 cytokines and polarize M2 macrophages in the context of type II diabetes in RAG-/- mice^131^. Despite the emerging roles of ILCs as antigen-presenting cells and key cellular regulators of mucosal immunity, how innate sensing in ILCs modulates their costimulatory properties is not clear. Further work is needed to understand how commensal microbial signals influence costimulatory and coinhibitory pathways in ILCs.

Similar to T cells, TLR2 can provide costimulation to ILCs. TLR2 signaling leads to increased LTi cell proliferation and IL-22 production^132^. The TLR2-MyD88 pathway is required for effective NK cell responses to vaccinia virus infection^133^. House dust mite extract activates TLR2 signaling in ILC2s, leading to increased production of IL-5 and IL-13^134^. On the other hand, treatment of ILC3s with LTA, another type of ligand for TLR2, depletes ILC3s by apoptosis^135^. Thus, TLR2 signaling can directly regulate the function and maintenance of ILCs, with differing outcomes depending on ligand type and ILC subset.

## Gut microbiota and immune checkpoint immunotherapy targeting coinhibitory pathways

One of the best examples of the association of gut microbiota and costimulation relates to immune checkpoint blockade cancer immunotherapies, one of the most successful cancer therapies. These therapies block PD-1, PD-L1, CTLA-4^136^ and other coinhibitory pathways. The gut microbiota has been clinically associated with these cancer immunotherapies in two ways.

First, the microbiota can modulate the efficacy of immune checkpoint blockade. Recent studies have shown the close association between gut microbial composition and responsiveness to anti-PD-1 cancer immunotherapy in cancer patients^39,137,138^. Initial studies demonstrated that different gut microbial species are enriched in patients who respond and those who do not respond to anti-PD-1 immunotherapy, and the same responses were reproduced in mice colonized with the patient microbiota. For example, *Akkermansia mucinophila* and *Enterococcus hirae* were found in responding patients, and mono-colonization of germ-free mice with these microbes induced a potent antitumor response to PD-1 blockade^39^. A defined consortium of gut microbes could induce potent responses to PD-1 blockade in tumor models^139^. More recently, fecal transplantation has been shown to overcome resistance to anti-PD-1 cancer immunotherapy, suggesting a causal relationship between anti-PD-1 therapy and the gut microbiota in patients^140,141^.

Despite ample evidence showing that the gut microbiota can regulate antitumor immune responses induced by immune checkpoint blockade, the mechanisms are not well understood. Several molecules secreted by the gut microbiota have been found to enhance the efficacy of immune checkpoint inhibitor therapy for cancer. Inosine, produced by *Bifidobacter pseudolongum*, was shown to promote the efficacy of anti-CTLA-4 by inducing TH1 responses through adenosine 2 A receptor signaling^11^. Enterococcus species secrete a hydrolase, SagA, which hydrolyzes peptidoglycan from the gut microbiota to promote an antitumor response in response to anti-PD-L1 via NOD2^142^. The gut microbiota can also release STING agonists to promote type 1 interferon production by monocytes and enhance antitumor responses by macrophages, DCs and NK cells^143^. Given that microbial signals control the expression of many costimulatory and coinhibitory molecules, gut microbes might also regulate responses to PD-1 inhibition by modulating other costimulatory and coinhibitory pathways. Our recent work showed that the gut microbiota can downregulate the PD-L2/RGMb axis and enhance antitumor immunity^144^. Indeed, many costimulatory and coinhibitory pathways interact with each other, and the efficacy of immune checkpoint blockade can be improved by the combined targeting of specific costimulatory and coinhibitory pathways^145^. Therefore, it is important to investigate how specific gut microbiota regulate costimulatory and coinhibitory pathways as a cancer immunotherapy response or resistance mechanism.

Second, immune-related adverse effects (IrAEs) that arise following immune checkpoint blockade have been correlated with microbiome composition^146,147^. Blocking PD-1, PD-L1 and CTLA-4 can result in these undesired inflammatory side effects^148^. Remarkably, a significantly high portion of patients with a high level of *Streptococcus sup*. developed irAEs after anti-PD-1 therapy^146^. Another study revealed that *Bacteroides intestinalis* and *Intestinalibacter Bartlettii* were enriched in a ≥grade 3 IrAE patient group, whereas patients who did not develop ≥grade 3 IrAE had microbiota enriched by *Anaerotignum lactatifermentas* and *Dorea formicigenerans*^149^. IL-1β and *B. intestinalis* are associated with combined immune checkpoint blockade-induced colitis in melanoma patients. The *Bacteroidetes* phylum is more prevalent in melanoma patients who are resistant to the development of anti-CTLA-4-associated colitis^150^, and metagenomic analyses in this study revealed an association of vitamin B biosynthesis pathways and polyamine transport with resistance to colitis development. Moreover, skin microbiota is linked to cutaneous IrAE^151^. IL-17A produced by T cells specific to the skin commensal microbe *Staphylococcus aureus* can mediate anti-CTLA-4-driven pathology in the skin. Impressively, a recent report showed that fecal transplantation from healthy donors to patients with immune checkpoint inhibitor-associated colitis completely resolved colitis symptoms^152^. Further work is needed to elucidate the mechanisms of microbiota-driven IrAEs.

## Conclusion

It is widely known that signals from gut microbiota play a pivotal role in shaping mucosal immune responses in the intestines. Commensal microbes produce various molecules recognized by the mucosal immune system. The recognition of commensal-derived molecules is mediated by canonical innate sensing mechanisms such as TLRs and NODs, which are used to detect pathogen-associated molecular patterns (PAMPs). Signal transduction through these pattern recognition receptors results in changes in the transcription of immune-related genes in innate cells. One of the significant changes in innate immune cells is an alteration in the capability of antigen-presenting cells to provide costimulation and coinhibition to the adaptive immune system. In this review, we focused on the relationship between microbe-derived signals and costimulatory and coinhibitory pathways. As ILCs are one of the most pronounced populations in mucosal sites, we discussed new insights into costimulation and immune regulation by ILCs. Importantly, ILCs can act as unconventional antigen-presenting cells that regulate mucosal T-cell responses through costimulatory pathways. Finally, we highlight the clinically relevant relationship between microbiota and costimulatory and coinhibitory pathways with an emphasis on response to immune checkpoint blockade. It is remarkable that the efficacy of immune checkpoint blockade therapies depends on the gut microbiota in patients. More surprisingly, distinct microbiota determine the development of IrAEs in the intestines and other barrier organs. This recent work illustrates how costimulation can mediate host-microbe interactions to shape the mucosal immune system. A deeper understanding of the mechanisms by which T-cell costimulatory and coinhibitory pathways mediate host-microbe interactions will provide insight into the regulation of mucosal immunity at homeostasis and during disease. In conclusion, we propose that innate sensing pathways and costimulatory and coinhibitory pathways in host immune cells closely cooperate with the microbiota to shape mucosal immunity.
